# Pre-warming the Streamlined Liner of the Pharynx Airway (SLIPA™) improves fitting to the laryngeal structure: a randomized, double-blind study

**DOI:** 10.1186/s12871-015-0151-4

**Published:** 2015-11-20

**Authors:** Hyun Kang, Dong Rim Kim, Yong Hun Jung, Chong Wha Baek, Yong Hee Park, Jong In Oh, Won Joong Kim, Geun Joo Choi

**Affiliations:** Department of Anesthesiology and Pain Medicine, Chung-Ang University College of Medicine, 224-1 Heukseok-dong, Dongjak-gu, Seoul 156-755 South Korea

**Keywords:** Pre-warming, SLIPA, Oropharyngeal leak pressure, Supraglottic airway devices

## Abstract

**Background:**

The Streamlined Liner of the Pharynx Airway (SLIPA), a type of supraglottic airway, has a non-inflatable cuff that softens at body temperature to fit the laryngeal structure. We investigated whether pre-warming of SLIPA to body temperature may improve insertion parameters.

**Methods:**

Ninety adult patients were assigned equally randomized to either Group W or Group R. Anesthesia was induced using propofol, fentanyl, and rocuronium. In Group W, the SLIPA was warmed to 37 ° C before insertion, whereas in Group R, it was inserted at room temperature. The insertion time, oropharyngeal leak pressure, postoperative throat pain, blood staining, regurgitation, number of attempts at insertion, and difficulty of insertion were compared between the two groups.

**Results:**

The insertion time was shorter in Group W than in Group R (3.60 [3.15–4.06] s vs. 6.00 [4.45–7.50] s; *P* < 0.001). Oropharyngeal leak pressure from the time of insertion until 3 min after insertion was significantly higher in Group W than in Group R (*P* < 0.05). Postoperative throat pain, measured using the visual analog scale, was lower in Group W than in Group R (0.00 [0.00–2.50] vs. 2.00 [0.00–4.50]; *P* = 0.006). The difficulty of insertion was lower in Group W than in Group R (*P* < 0.004). There were no significant differences in terms of blood staining, regurgitation, and number of attempts.

**Conclusions:**

Pre-warming the SLIPA to body temperature has significant benefits compared to maintaining the device at room temperature. Specifically, insertion was easier, both insertion and fitting to the laryngeal structure could be performed more quickly, and the incidence of sore throat was reduced.

**Trial registration:**

Clinical Research Information Identifier NCT01209000

## Background

Supraglottic airway devices (SADs) are widely used, both in emergencies to quickly secure the airway and in elective surgery to reliably ventilate the airway during both spontaneous and assisted breathing. The Streamlined Liner of the Pharynx Airway (SLIPA™), a second-generation SAD, has theoretical advantages over first-generation SADs because it has potentially lower risk of aspiration due to the large capacity of the chamber (50mL), even when regurgitation occurs, and its ability to provide a better perilaryngeal seal [1, 2]. The SLIPA is cuffless, negating the need for inflating and controlling the pressure of the cuff.

The SLIPA is composed of a thermoplastic material (polyethylene and vinyl acetate), and it was designed to increase the effectiveness of sealing after insertion. The stiffness of the SLIPA is reduced at body temperature, providing a better fit to the perilaryngeal structure [3]. Based on this property, we thought that pre-warming the SLIPA to body temperature (37 ° C) before insertion might induce the sealing effect more quickly. However, no prior study investigated the effects of pre-warming the SLIPA, and thus, we designed this study.

In addition, the SLIPA is stiffer than other SADs, and this leads to an increase in the incidence of blood staining, an indicator of direct trauma. However, postoperative throat pain is not increased [4]. We also assumed that by pre-warming the SLIPA, we would lower the incidence of blood staining and diminish the severity of throat pain as a result of the softening effect.

The purpose of this study was to compare two SLIPA insertion methods. In one method, the SLIPA was kept at room temperature before insertion, and in the other, the SLIPA was pre-warmed to body temperature (37 ° C) before insertion. We evaluated oropharyngeal leak pressure (OLP), insertion time, postoperative throat pain, and difficulty of insertion in both groups.

## Methods

### Study protocol and patients

The protocol of this study was approved by the Institutional Review Board of Chung-96 Ang University Hospital [C2014020(1216)] and registered at Clinical Research Information Service [KCT0001059]. The study was conducted according to the principles of the 2000 revision of the Declaration of Helsinki, and written informed consent was obtained from all patients.

This study was intended for American Society of Anesthesiologists (ASA) class I-II patients aged 20 to 65 years who received general anesthesia for elective gynecologic, orthopedic, or abdominal surgery performed in the supine position and lasting for <2 h. The exclusion criteria were as follows: body weight of <40 kg or >100 kg, body mass index > 30 kg/m^2^, pregnancy or lactation, a history of upper abdominal surgery, diabetes mellitus, hiatus hernia and gastroesophageal reflux disease, peptic ulcer, risk of aspiration or regurgitation, and concomitant serious cardiovascular, pulmonary, renal, hepatic, hematologic, or cervical disease. The decision to enroll or exclude patients was made by the principle investigator, who did not otherwise participate in the study and data collection.

### Study design and randomization

This was a randomized, double-blind study. Randomization into one of two groups was based on a table generated using PASS 11™ software (NCSS, Kaysville, Utah, USA). Details of the series, which was generated by a statistician who did not otherwise participate in the study, were unknown to both the investigators and patients, and the numbers were contained in a set of sealed envelopes. Fifteen minutes before patients were admitted to the operating room, the appropriate numbered envelope was opened, and the card inside determined whether the patient was assigned to Group W (warming group) or Group R (room temperature group). The investigator who read the card prepared the SLIPA at either body or room temperature. For Group R, the SLIPA was immersed in water at room temperature (22 ° C) 15 min before insertion, whereas for Group W, it was immersed in warmed (37 ° C) water, which was kept in a heating cabinet set to 37 ° C.

To blind the anesthesiologist who inserted the SLIPA to its temperature, he wore both cotton and PVC gloves.

### Anaesthesia

The patients fasted from midnight on the day of surgery, and no premedication was administered before anesthesia. After the placement of standard monitoring systems (electrocardiograph, non-invasive arterial blood pressure sensor, pulse oximeter), each patient was denitrogenated with 100 % oxygen. Anesthesia was induced with intravenous fentanyl (2 μg/kg), lidocaine (0.5 mg/kg), and propofol (2 mg/kg). After confirming that the patient had become unconscious and corneal reflex had disappeared, rocuronium (0.6 mg/kg) was administered, and manual ventilation with sevoflurane (3 vol. %) in 100 % O_2_ (5 L/min) was simultaneously performed. After the peripheral nerve stimulator (NMT MechanoSensor, GE Health Care Filand Oy, Helsinki, Finland) with electrodes placed over the ulnar nerve revealed that the count of twitches of the train-of-four stimulation reached zero, the appropriately prepared SLIPA was inserted. The size of the SLIPA was chosen by matching the width the thyroid cartilage with that of the bridge of the SLIPA [5]. First, a sniffing position was made by placing a 5-cm pillow under the patient’s head, and the bridge area of the SLIPA was collapsed. One hand lifted the patient’s jaw, whereas the other hand pushed the SLIPA into position so that the heel portion of the SLIPA was in the nasopharynx over the base of the tongue. The insertion was performed by a single anesthesiologist with more than 3 years of experience in SLIPA insertion.

When insertions were attempted more than twice without success, tracheal intubation was performed, and these patients were excluded from the study.

The time taken to complete the insertion, assessed from the touching of the SLIPA to the teeth to its fixation to the laryngeal structure, was measured. The anesthesiologist who inserted the SLIPA subjectively evaluated the difficulty of the insertion (easy, normal, or hard).

Successful insertion was confirmed by uniform movement of both lungs, a normal capnogram, and a peak inspiratory airway pressure of <30 cmH_2_O [6]. After insertion, OLP was immediately measured, and this measurement was repeated at 1-min intervals for 5 min. After converting to the manual ventilation mode and setting the adjustable pressure limiting valve to maximum, OLP was measured when the leakage sound began to be heard through a stethoscope placed over the patient’s mouth. If the sound was not heard until 40 cmH_2_O, OLP was recorded as >40 cmH_2_O.

The mechanical ventilation settings were as follows: tidal volume of 6–8 mL/kg of the patient’s ideal body weight, an inspiratory-to-expiratory ratio of 1:2, fixed using a volume-controlled ventilator (Datex-Ohmeda Aestiva/5™, GE Health Care, Madison, WI, USA), and a respiratory rate of 10–12/min.

Anesthesia was maintained with desflurane (6–10 vol. %) and 60 % N_2_O (3 L/min). All surgeries were performed with patients in the supine position.

At the end of surgery, following the return of spontaneous breathing and if the patient could obey the commands, the SLIPA was removed. We examined the device for the presence of blood staining or evidence of gastric reflux. The severity of postoperative sore throat was determined using the visual analog scale (VAS) in the recovery room after at least 30 min. A second anesthesiologist who did not insert the SLIPA and who had been blinded to the patients’ assignment to groups recorded postoperative data as an independent observer.

### Statistical analysis

The primary outcome measurement of the study was OLP, measured immediately and 1, 2, 3, 4, and 5 min after insertion. To estimate the necessary group size for the study, a pilot study was conducted to measure OLP in 10 patients in whom the SLIPA was inserted at room temperature. The average OLPs immediately after insertion and 1, 2, 3, 4, and 5 min after insertion were 22.0, 24.1, 24.7, 26.3, 27.0, and 28.9 mmHg, respectively. The standard deviations of OLP ranged from 4.1 to 6.5, and an autocorrelation between adjacent measurements on the same individual of 0.7 was found. For our power calculation, we assumed that first-order autocorrelation adequately represented the autocorrelation pattern. Thus, it was necessary to detect a 10 % higher OLP in Group W than in Group R. With an α value of 0.05 and a power of 80 %, 42 patients were required for each group. Considering a likely insertion failure or dropout rate of 5 %, 90 patients were required for the study. PASS 11™ software (NCSS, Kaysville, UT, USA) was used to calculate the necessary sample size.

For continuous variables, the normal distribution of the collected data was first evaluated using the Shapiro-Wilk test. Normally distributed data were presented as the mean ± standard deviation, and groups were compared using the unpaired t-test. Non-normally distributed data were expressed as medians (25th percentile–75th percentile) and were analyzed using the Mann–Whitney U test.

As OLP did not pass the Shapiro-Wilk test, we additionally checked the q-q plot, which did not show marked deviation from linearity. Therefore, we decided to apply the normal assumptions for the repeated-measured analysis of variance (ANOVA) in the analysis of OLP. Because the sphericity assumptions failed, we used multivariate analysis of variance (MANOVA), followed by a *t*-test with Bonferroni’s correction. The descriptive variables were analyzed by either Chi-squared analysis or Fisher’s exact test, as appropriate. *P* < 0.05 was considered statistically significant. Statistical analysis was performed using SPSS version 18.0 (IBM Corp., Armonk, NY, USA).

## Results

**Fig. 1:**
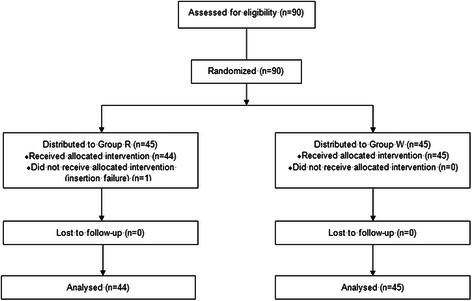
CONSORT flow diagram. The progress of patients through the trial is shown. Group R: the Streamlined Liner of the Pharynx Airway (SLIPA) was kept at room temperature; Group W: the SLIPA was pre-warmed to 37 ° C

Of the 90 patients who were recruited for this study between April 2014 and December 2014, 45 patients each were randomized into Groups W and R. There were no differences between the groups in terms of age, gender, height, weight, ASA class, the Mallampati score, and the duration of anesthesia (Table 1). One insertion failure (1/45) occurred in Group R. There were no insertion failures in group W (Fig. 1).

**Table 1:** Demographic data

	Group R (*n* = 44)	Group W (*n* = 45)	P
Age (years)	41.3 ± 12.5	37.4 ± 11.6	0.130
Sex (male/female)	16/28	18/27	0.724
Height (cm)	163 (159–169)	162 (159–171)	0.583*
Weight (kg)	64.2 ± 11.3	63.6 ± 10.8	0.800
ASA (1/2/3/4)			
Mallampati score (1/2/3/4)	9/11/13/11	9/16/6/14	0.278
Duration of anesthesia (min)	78.7 ± 31.3	72.9 ± 29.1	0.361

Values are expressed as means ± standard deviations, medians (25th percentile–75th percentile), or absolute numbers

*The Mann–Whitney U-test was used, and the data were expressed as medians (25th percentile–75th percentile) because of the abnormal distribution

**Fig. 2:**
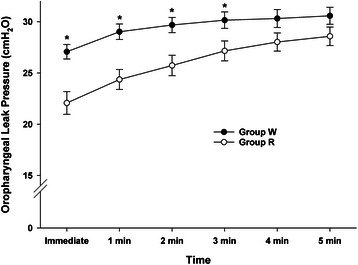
Oropharyngeal leak pressure (OLP). OLP increased gradually over time in both groups. However, the mean OLP in Group W was significantly higher than that in Group R from the point of insertion until 3 min after insertion. Group R: the Streamlined Liner of the Pharynx Airway (SLIPA) was kept at room temperature; Group W: the SLIPA was pre-warmed to 37 ° C. Values are expressed as the mean ± SE. *P < 0.05

The results for OLP are shown in Fig. 1. OLP increased gradually over time in both groups. However, the mean OLP in Group W was significantly higher than that in Group R from the point of insertion until 3 min after insertion (*P* < 0.05). Four minutes after insertion of the SLIPA, there was no longer any significant difference between the two groups concerning OLP (Fig. 2).

**Fig. 3:**
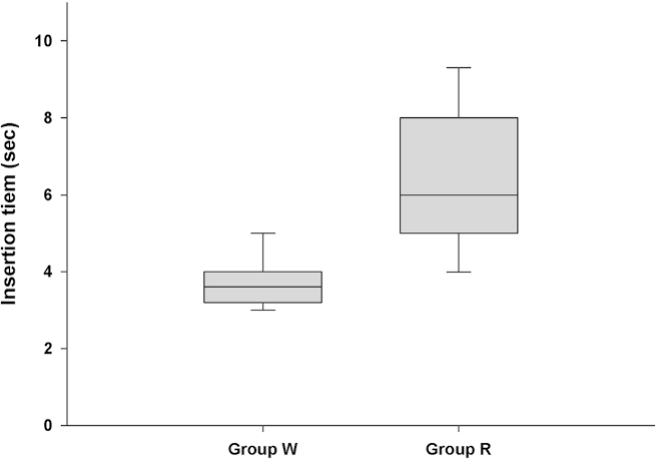
Insertion time. The insertion time was faster in Group W (3.60 s [3.15–4.06 s]) than in Group R. Box-and-whisker plot (median, interquartile range, and range) of the insertion time in Group R and Group W. Group R: the Streamlined Liner of the Pharynx Airway (SLIPA) was kept at room temperature; Group W: the SLIPA was pre-warmed to 37 ° C. *P < 0.001

The insertion time was faster in Group W (3.60 s [3.15–4.06 s]) than in Group R (6.00 s [4.45–7.50 s]; *P* < 0.001; Fig. 3).

The insertion was easier in Group W than in Group R (*P* = 0.004). There were no significant differences in terms of blood staining, regurgitation, and the number of attempts (Table 2).

**Table 2:** Airway observations

	Group R (*n* = 44)	Group W (*n* = 45)	P
Blood staining (n)	5	4	0.699
Regurgitation (n)	0	0	NA
Attempt (1/2; n)	44/0	45/0	NA
Difficulty of insertion (easy/normal/hard)	7/28/9	21/21/3	0.004*

Values are expressed as absolute numbers

*P < 0.05 between groups

**Fig. 4:**
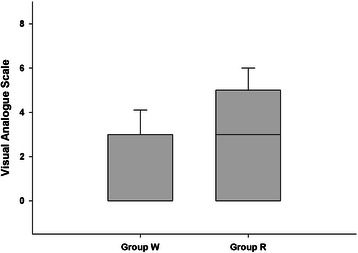
Postoperative throat pain (visual analog scale [VAS]). Postoperative throat pain, determined using the VAS, was lower in Group W than in Group R. Box-and-whisker plot (median, interquartile range, and range) of the VAS in Group R and Group W. Group R: the Streamlined Liner of the Pharynx Airway (SLIPA) was kept at room temperature; Group W group: the SLIPA was pre-warmed to 37 ° C. *P = 0.006

Postoperative throat pain, determined using the VAS, was less severe in Group W than in Group R (0.00 [0.00–2.50] vs. 2.00 [0.00–4.50]; *P* = 0.006; Fig. 4).

## Discussion

We demonstrated that pre-warming the SLIPA to 37 ° C, opposed to using it at room temperature, results in quicker sealing to the perilaryngeal structure. This is evidenced by the fact that OLP was higher immediately after insertion when a warmed SLIPA was used, and this remained the case until 3 min after insertion. We also found that the severity of postoperative throat pain, insertion time, and difficulty of insertion were all significantly reduced.

SADs exhibit excellent hemodynamic stability during insertion in comparison with endotracheal intubation [7, 8]. Furthermore, insertion is easier and faster [9, 10]. In addition, because SADs are positioned above the glottis, opposed to the trachea, without passing through the vocal cords, they are associated with fewer airway complications such as emergence laryngospasm, coughing, postoperative hoarseness, and throat pain. For this reason, SADs have been used frequently during general anesthesia [11, 12]. However, serious complications, such as SADs twisting or blocking the airway, also occur. In such cases, it is necessary to switch to tracheal intubation [13–15]. In addition, there is a fear of pulmonary aspiration caused by regurgitation. However, according to previous research, SAD insertion was associated with no difference in the risk of pulmonary aspiration caused by regurgitation compared with endotracheal intubation among people who did not have a disease that increases the risk of regurgitation [11, 13, 15–17].

The SLIPA, a new-generation SAD, is classified as a cuffless, anatomically pre-shaped base of the tongue sealer [18]. It has a hollow chamber of approximately 50 mL to prevent aspiration pneumonia caused by regurgitation, which can occur when using an SAD. Otherwise, i-gel have separated gastric drainage channel that prevent to aspiration. However, if the drainage channel is blocked by particulate matter, the advantages are decreased. In addition, there is a risk of laryngospasm if the suction catheter contacts the glottis while passing through the distal opening [19]. Although there is no cuff, the SLIPA is composed of a thermoplastic elastomer (polyethylene and vinyl acetate). Once inserted, the rigid material is softened at body temperature, and it can therefore effectively seal to the perilaryngeal structure. Interestingly, in a previous study, although the i-gel, Supreme LMA, and ProSeal LMA consist of different materials, the OLPs associated with these devices improved over time [20]. In addition, the heel portion of the SLIPA, which is placed in the nasopharynx, provides higher OLP. As a result, the SLIPA can be used in laparoscopic surgery demanding high airway sealing pressure, and it is also stable during changes in operation position [1, 6, 21, 22].

OLP is an effective indicator for assessing the success of SADs in protecting the airway and providing positive pressure ventilation [23]. According to a previous study, despite the lack of an inflatable cuff, the SLIPA is associated with a similar OLP as other SADs because of its resemblance to the perilaryngeal structure [1]. We found that pre-warming the SLIPA results in a higher OLP than the previous method of room-temperature insertion for up to 3 min. With faster sealing, a more reliable positive pressure ventilation is available, and the risk of aspiration can be further reduced.

The i-gel and SLIPA are classified as cuffless SADs. The i-gel is also composed of a thermoplastic elastomer (styrene ethylene butadiene styrene), and it is warmed by body temperature after insertion. It can also fit more efficiently to the perilaryngeal structure in the same manner as the SLIPA [24]. There have been several studies of pre-warming of the i-gel before insertion. Among them, one study that utilized pre-warming to 37 ° C did not uncover statistically significant results [25]. However, a study utilizing pre-warming to 42 ° C reported more successful positive pressure ventilation because of the achievement of a higher OLP. Komasawa et al. reported that the reason for the reduced sealing effect in the study with pre-warming to 37 ° C was decreasing temperature during insertion [26]. In both studies, the i-gel was stored in the heating cabinet for 30 min before insertion. However, because of the rather large volume of the thermoplastic elastomer in one part of the i-gel, this heating time may not have been sufficient. On the contrary, because the SLIPA is composed of a relatively thin thermoplastic elastomer, we were able to successfully improve positive pressure ventilation via incubation in a heating cabinet set to 37 ° C for only 15 min before insertion.

By pre-warming the SLIPA, the insertion time was significantly decreased, and the maneuver was made easier. This was the case because the fairly rigid SLIPA was made softer after warming to body temperature.

The severity of postoperative throat pain was reduced by pre-warming the SLIPA, but the incidence of blood staining did not differ between the two groups. We expected that as an indicator of a direct trauma to the pharyngeal mucosa, the incidence of blood staining also would be further reduced by pre-warming the SLIPA; however, the incidence was similar between the groups, in line with the findings of previous research [4]. In addition, pre-warming the SLIPA slightly softens the device, resulting in reduced throat pain, but the incidence of blood staining was not reduced because of the relatively large chamber of the SLIPA for preventing aspiration by regurgitation.

Our study has some limitations. First, our results may have been influenced by the methods of the size selection of the SLIPA. When selecting the appropriate SLIPA size, some methods are based on thyroid cartilage width, whereas others are based on height and gender. We selected SLIPA size based on the thyroid cartilage width because it is known that this is the more efficient method [5]. Of course, if we had selected SLIPA size based on height and gender, the results may have been different. Second, we did not measure leak volume. The leak volume is calculated as the inspiratory volume minus the expiratory volume. It is measured during mechanical ventilation, whereas the OLP is measured during manual ventilation. Therefore, we could not measure the leak volume when we checked the OLP. Third, this study was conducted at a single institution. A multicenter study may have more clearly demonstrated the usefulness of pre-warming the SLIPA.

## Conclusions

In conclusion, pre-warming the SLIPA to body temperature has significant benefits compared to maintaining the device at room temperature because pre-warming the SLIPA can ease the insertion, shorten the time needed for both insertion and fitting to the laryngeal structure, and reduce the incidence of sore throat. The initial OLPs were higher in the pre-warmed group; however, after 3 min, there was no significant difference between the groups.

## Abbreviations

SAD: Supraglottic airway device
SLIPA: Streamlined Liner of the Pharynx Airway
VAS: Visual analog scale
OLP: Oropharyngeal leakage pressure
ASA: American Society of Anesthesiologists
